# The chromosome-level genome and functional database accelerate research about biosynthesis of secondary metabolites in *Rosa roxburghii*

**DOI:** 10.1186/s12870-024-05109-1

**Published:** 2024-05-17

**Authors:** Jiaotong Yang, Jingjie Zhang, Hengyu Yan, Xin Yi, Qi Pan, Yahua Liu, Mian Zhang, Jun Li, Qiaoqiao Xiao

**Affiliations:** 1 Resource Institute for Chinese and Ethnic Materia Medica, Guizhou University of Traditional Chinese Medicine, Guizhou, 550025 China; 2 College of Agronomy, Qingdao Agricultural University, Qingdao, 266109 China; 3 State Key Laboratory of Plant Diversity and Specialty Crops, Institute of Botany, The Chinese Academy of Sciences, Beijing, China

**Keywords:** *R. Roxburghii*, Genome, Functional annotation, Database, Metabolites biosynthesis

## Abstract

*Rosa roxburghii* Tratt, a valuable plant in China with long history, is famous for its fruit. It possesses various secondary metabolites, such as L-ascorbic acid (vitamin C), alkaloids and poly saccharides, which make it a high nutritional and medicinal value. Here we characterized the chromosome-level genome sequence of *R. roxburghii*, comprising seven pseudo-chromosomes with a total size of 531 Mb and a heterozygosity of 0.25%. We also annotated 45,226 coding gene loci after masking repeat elements. Orthologs for 90.1% of the Complete Single-Copy BUSCOs were found in the *R. roxburghii* annotation. By aligning with protein sequences from public platform, we annotated 85.89% genes from *R. roxburghii*. Comparative genomic analysis revealed that *R. roxburghii* diverged from *Rosa chinensis* approximately 5.58 to 13.17 million years ago, and no whole-genome duplication event occurred after the divergence from eudicots. To fully utilize this genomic resource, we constructed a genomic database RroFGD with various analysis tools. Otherwise, 69 enzyme genes involved in L-ascorbate biosynthesis were identified and a key enzyme in the biosynthesis of vitamin C, GDH (L-Gal-1-dehydrogenase), is used as an example to introduce the functions of the database. This genome and database will facilitate the future investigations into gene function and molecular breeding in *R. roxburghii*.

**Supplementary Information:**

The online version contains supplementary material available at 10.1186/s12870-024-05109-1.

## Introduction

*Rosa roxburghii* Tratt, which belongs to Rosaceae family, is a wild deciduous, perennial shrub. It is also named Cili in China because its golden fruit covered with tiny prickles. The fruit of *R. roxburghii* has a faint aroma with a bit sour and astringent taste, but it is famous for its nutritious and medical function [1]. *R. roxburghii* is wildly distributed in the alpine and hilly areas of southwest China, especially in Guizhou province with large temperature difference [2]. In China, in addition to wild *R. roxburghii*, several germplasm resources are cultured artificially, such as ‘Guinong 5’ (Rr-5) [3]. Since the medicinal and commercial value are discovered, the research on *R. roxburghii* has been more and more popular. *R. roxburghii* fruits contain various nutrients, such as carbohydrates, amino acids, vitamins, proteins, minerals and dietary fibers, which are benefit to our health [2, 4]. Otherwise, active ingredients including superoxide dismutase (SOD), organic acids, polysaccharides, flavonoids, polyphenols, triterpenoids, glycosides etc., play important roles in medical function [2]. Several research have demonstrated that *R. roxburghii* poses the function of antioxidant, anti-tumor, anti-inflammatory, anti-radiation, anti-diabetes, anti-radiation and anti-aging [5–9]. All these functions depend on the active ingredients in the plant. Furthermore, *R. roxburghii* is widely used in the food industry, such as herbal tea, jam, vinegar, yoghurt and moon cake, which take *R. roxburghii* as raw materials to enhance the flavor of food [10]. Wide application of *R. roxburghii* in various field makes it a promising crop with broad market prospects.

Recently, long-read sequencing technology and high-throughput chromosome conformation capture technology make it possible to obtain the genome sequences of various plants [10, 11]. Pu et al. assembled the chromosome level genome of *Lonicera japonica* [12]. Combination of genome and transcriptome analysis, they elucidated the molecular mechanism of dynamic flower color, which provided valuable genetic resources for molecular breeding and important clues for the evolution of *L. japonica* family [12]. Tu et al. constructed a high-quality *Tripterygium wilfordii* genome and found that cytochrome P450 participated in the metabolism of triptolide [13]. This important study contributed to elucidation the pathway of triptolide biosynthesis and further laid the foundation for the heterologous bioproduction of triptolide [13]. Zhang et al. completed whole genome assembly of *Dendrobium chrysotoxum* and comparative genomic analysis revealed molecular regulatory mechanisms such as medicinal components, flower color, flowering duration and stress tolerance, providing genetic basis for the medicinal and horticultural development of this plant [14]. Jiang et al. deciphered haplotype-resolved genome of *Bletilla striata* and revealed the mechanism of *B. striata* polysaccharide (BSP) biosynthesis [15]. Comparative genomic analysis further indicated that the expansion of *B. striata* gene family may played an important role in secondary metabolite biosynthesis and environmental adaptation [15]. These chromosome-scale genome information provided the basis for the heredity and evolution of species, and also laid the foundation for the study of gene function. However, there is a lack of a functional genomic database and its associated applications based on a high-quality genome assembly of *R.roxburghii*.

In our study, we assembled a chromosome-scale genome of *R. roxburghii*. Combined with genome and transcription data, we constructed a gene functional database, named RroFGD (http://www.gzybioinformatics.cn/RroFGD). In addition, we integrated basic local alignment search tool (Blast), extract sequence, gene set enrichment analysis (GSEA), heatmap and JBrowse analysis tools into the database for mining gene function. Using the database, we identified 69 key enzyme genes involved in ascorbate biosynthesis based on KEGG annotation information. We also took a vitamin C synthetase RrGDH as an example to introduce the functions of the database. The analysis results indicated that *RrGDH* gene might play an important role in the biosynthesis of L-ascorbate. The genome and database revealed here will provide reference not just for comprehension of *R. roxburghii* evolution but also for mining the gene function.

## Materials and methods

### Plant materials

**Fig. 1:**
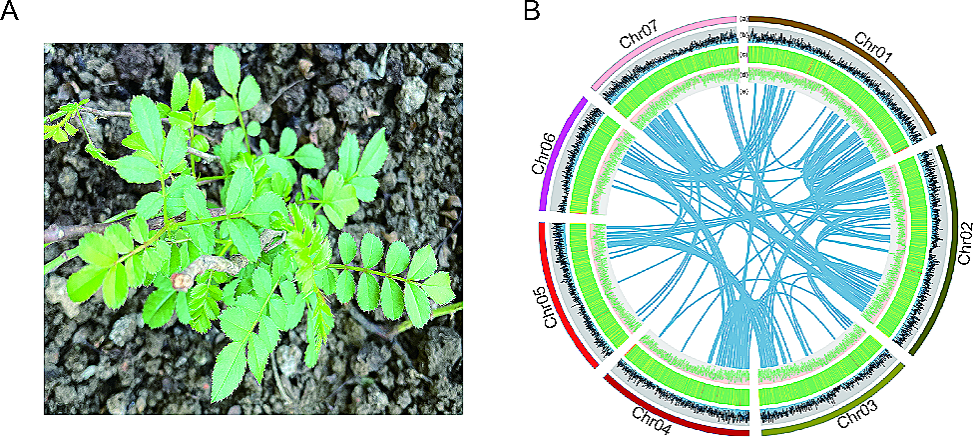
*R. roxburghii* plant used for sequencing, assembled genome features, and synteny information. (**A**) *R. roxburghii* plant. (**B**) a represents chromosome number and relative length, b represents gene density in different chromosomes, c represents GC density in the genome, d represents repeat element density, and e represents synteny blocks of paralogous sequences

Plant materials of *R. roxburghii* seedling were collected in the wild of Hongfeng Lake Scenic Area, Qingzhen City, Guizhou, China (Fig. 1A). The voucher specimen has been identified as *R. roxburghii* Tratt by Professor Chenggang Hu at Guizhou University of Traditional Chinese Medicine and is deposited at the Miao Medicine Museum of Guizhou University of Traditional Chinese Medicine (Collection Number: GZTM0220111).

### Illumina and ONT sequencing

We collected all the samples from an individual R. roxburghii. The sequencing data comprised four main components. The first part was second-generation DNA sequencing data, utilized for genome size assessment, which were sequenced using the illumina platform on leaf DNA. The second part was HIC data, employed for aiding genome scaffolding, also were sequenced using the illumina platform on leaf DNA. The third part was third-generation DNA sequencing data, utilized for genome assembly, which were sequenced using the ONT platform on high-quality leaf DNA. The fourth part was third-generation full-length transcriptome data, utilized for assisting gene structure annotation, which were sequenced using the ONT platform on high-quality RNA from mixed tissues including roots, stems, and leaves. All sequencing was performed in the Wuhan Bebagene Technology Co., Ltd (Wuhan, China).

### Genome survey and assembly

After obtaining the illumina paired-end (2 × 150 bp) sequencing data, we measured the k-mer profile (k = 19) according to clean reads by Jellyfish software [16]. Genome size and heterozygosity were predicted basing on the k-mer profile by gce software (v1.0.2) [17]. ONT reads were directly assembled using necat software [18]. The assembled contigs were polished four times by nextPolish software (v1.3.1) [19], using illumina short reads.

### Chromosomal genome assembly and chromatin interactions through Hi-C technology

First, *R. roxburghii* leaves were crosslinked using 1% formaldehyde. Then, cells were lysed using Dounce homogenizer method to release chromosomes and DNA. DNA was digested using DPNII, generating sticky ends. These ends were filled and labeled with biotin using DNA polymerase. The filled DNA ends were then ligated to form linked products. The biotin labeled Hi-C linked products were pulled down to generate a library suitable for sequencing. Finally, the library was sequenced using illumina sequencing technology. The contigs were clustered and arranged on pre-chromosomes based on the predicted intrachromosomal interaction information using 3d-DNA software [20]. Then we manually corrected the assembly using the Juicer-box software and recalculated interaction matrix of HIC by HiC-Pro [21]. Finally, the chromatin interaction heat map was drawn by HiCpotter [22]. The completeness of the genome assembly was assessed using Benchmarking Universal Single-Copy Orthologs (BUSCO) with the Embryophya odb 10 dataset (BUSCO; v5.2.2) [23].

### Genome annotation

RepeatModeler (v2.0.1) [24] and RepeatMasker (v4.1.0) [25] were used to detect and annotate repeated elements within the genome of *R. roxburghii*. Next, we used second-generation, third-generation, homologous alignment evidence, and de novo prediction methods to annotate the gene structure of genome. Specific steps are as follows: (1) We firstly used second-generation transcriptome data (SRA number SRP167314) including leaves, stems, flowers and fruits for gene structure prediction. Then the transcriptome was aligned to the genome using the software hisat2 (version 2.1.0) [26], and the resulting transcript was reconstructed by stringtie (version 2.1.4) [27]. We further used the software TransDecoder (version: v5.1.0) to predict coding box of the predicted transcript region, and obtained the coding genes of the second-generation transcriptome prediction. (2) For the full-length transcriptome annotation, we used the software NanoFilt (version: 2.8.0) to conduct data filtering and the software Pychopper (version: v2.7.2) for full-length sequence identification. The resulting full-length sequence was self-corrected using racon (version: v1.4.21) [28] based on the original reads. We compared the corrected full-length sequence with the genome using minimap2 (version 2.17-r941) [29] and reconstructed transcript by stringtie (version 2.1.4). The software TransDecoder is used to predict the coding box of the predicted transcript region, and finally the predicted coding genes were obtained. (3) The homologous protein sequences were compared to the genome using the software tblastn [30], and then Exonerate (version v2.4.0) [31] was used to predict the transcript and coding region based on the comparison results. (4) Gene structures predicted by the second-generation transcriptome were performed model training and de novo prediction by Augustus (version: 3.3.2). (5) MAKER (v2.31.9) [32] was used to integrate the genetic annotation results predicted by various software.

For gene functional annotation, we aligned *R. roxburghii* protein sequence against protein databases such as Nr, Uniprot, Swissprot, and TAIR by diamond blastp software [33]. The Uniprot database recorded the protein families and their corresponding Gene Ontology (GO) annotations. Using the annotation information from the Uniprot database, GO annotations of the corresponding genes were extracted. The sequences were aligned against the KEGG database by diamond blastp. Then, KOBAS [34] was used to combined the sequences with KEGG Orthology and Pathway information based on ID mapping. The completeness of the genome annotation was also assessed using Benchmarking Universal Single-Copy Orthologs (BUSCO) with the Embryophya odb 10 dataset (BUSCO; v5.2.2) [23].

### Genome evolution analysis

Orthfinder [35] was used to cluster the amino acid sequences of *R. roxburghii* and 13 other angiosperms into orthologous groups. Using the RAxML package (v 8.1.13) [36], a maximum likelihood phylogenetic tree was constructed according to single-copy genes obtained from *R. roxburghii* and the 13 other angiosperms. Divergence time were estimated by the mcmctree program in the PAML software package [37]. The CAFÉ software (v5) [38] was used to detect gene family expansions and contractions. WGD software [39] was used to calculate the synonymous substitutions per synonymous site (ks) values between *R. roxburghii* and *R. roxburghii*, *R. roxburghii* and *A.thinana*, *R. roxburghii* and *Glycine max, R. roxburghii* and *Malus domestica, R. roxburghii* and *Fragatia vesca*. Syntenic blocks between *R. roxburghi* and *R. roxburghii, R. roxburghii* and *Rose rugosa, R. roxburghii* and *Rose Chinensis* were identified using MCScanX software [40]. Furthermore, we constructed a Circos map by Circos v0.52 [41] to display the genome information of *R. roxburghii*.

### Construction of co-expression network

We downloaded transcriptome data samples from the SRA database and used the Hisat2 software [26] to map the downloaded transcriptome data to the reference genome of *R. roxburghii*. Then, we used stringtie software [42] to obtain the TPM values of each transcriptome sample and constructed an expression matrix. We calculated the correlation between gene expressions for every pair of genes using the PCC algorithm. After that, we ranked the gene correlations using the MR algorithm. Finally, we evaluated the network using ROC curves and selected an appropriate threshold to construct a co-expression network. The formula is as follows:

$$PCC=\frac{\sum (X-\stackrel{-}{X})(Y-\stackrel{-}{Y})}{\sqrt{{\sum }_{i=1}^{n}{({X}_{i}-\stackrel{-}{X})}^{2}}\sqrt{{\sum }_{i=1}^{n}{({Y}_{i}-\stackrel{-}{Y})}^{2}}}$$

$$MR\left(AB\right)=\sqrt{Rank\left(AB\right)\times Rank\left(BA\right)}$$

#### Note

‘n’ represents the total number of samples present in the RNA-seq data. The variables ‘x’ and ‘y’ represent the TPM values. The term ‘Rank’ denotes the position of PCC values. Specifically, ‘AB’ signifies the ranking of gene A among all genes when compared to gene B, while ‘BA’ indicates the reverse ranking, i.e., the position of gene B when compared to gene A.

For assessment the reliability of network and establishment specific threshold values of both PCC and MR metrics, we identified Gene Ontology (GO) terms related to biological processes, specifically focusing on those with gene counts ranging from 4 to 20, which were designated as prior gene sets. Additionally, we selected co-expressed genes under the defined threshold to form other gene sets. Whether GO could be accurately in co-expression network gene pairs was used as input for a specific binary classifier and calculated the true positive rate (TPR) and false positive rate (FPR), then plotted the ROC curve. By comparing the sizes under the ROC curve (AUC) at various thresholds, we determined MR values that yielded the maximum AUC, representing the optimal cutoff to define the co-expression network.

### Protein-protein interaction (PPI) network

The OrthoFinder software [35] was used to predict orthologous relationships between *Arabidopsis* and *R. roxburghii*. Subsequently, PPI network was mapped from *Arabidopsis* to *R. roxburghii*, establishing the PPI network in *R. roxburghii*.

### Gene family identification

OrthoFinder [35] was used to predict the orthologous relationship of proteins between *Arabidopsis* and *R. roxburghii*. Subsequently, CAZy and TP were identified based on this orthologous relationship. The iTAK software [43] was utilized to identify and classify transcription factors, transcription regulators and protein kinases in *R. roxburghii*. A hidden Markov model obtained from iUUCD 2.0 [44] was used to identify ubiquitin families successfully in *R. roxburghii*. Functional annotation of CYP450 genes was performed based on the KEGG annotations.

### Construction of RroFGD

Based on the LAMP (Linux, Apache, MySQL, PHP) technical stack, *R. roxburghii* functional genomics database was built. A MySQL database was created by importing various results and data analyses, such as gene structure annotations, gene functional annotations, co-expression network, PPI network, and gene family classification. To enhance data visualization and analysis, dynamic websites were developed using HTML, PHP, JavaScript, and CSS languages.

### Toolkit for gene function analysis

We integrated Gene Set Enrichment Analysis (GSEA) [45] as previous descriptions [46–48]. ViroBlast [49] was used to achieve the sequence alignment function on line. We also incorporated JBrowse software [50] to display gene structure and RNA-seq mapping states. Furthermore, we introduced a sequence extraction tool using a Perl script and implemented a Heatmap analysis tool based on Highchart Javascript. These additions expanded the capabilities of the platform and improved the visualization and analysis of data.

### Identification and analysis of genes involved in L-ascorbate biosynthesis

The L-ascorbate biosynthesis pathway involved 18 gene families, including HK, PGI, PMI, PMM, GMP, GME, GGP, GPP, GDH, GLDH, MDHAR, DHA, APX, GalUR, GulLO, ALase, GulDH and MIOX genes. To identify candidate genes related to the L-ascorbate biosynthesis pathways in *R. roxburghii* genome, we screened the data based on functional annotation information from the KEGG. According to co-expression network analysis, we identified transcription factors that showed co-expression relationships with the key enzyme genes. In addition, we selected apple, which belonged to the *Rosaceae* family as the input species and analyzed the binding sites of key enzyme gene promoters using the online tool PlantRegMap [51].

## Results

### Genome sequencing and assembly of *R. Roxburghii*

The genome size of *R. roxburghii* was estimated to be 497.8 Mb by 19 k-mer distribution analysis, with a heterozygosity of 0.25% (Figure S1). Using the Oxford Nanopore PromethION Sequencer, we obtained 50.79Gb of ONT reads with an N50 of 34.8 kb by sequencing and the genome coverage was about 100×. ONT reads were subjected to assemble using necat software [18], the assembled contigs underwent four rounds of polishing using illumina short reads via nextPolish (v1.3.1) [19], leading to the construction of a scaffold assembly with an N50 of 13 Mb. The global mapping rate of the DNA illumina sequencing reads to the assembled reference genome was 93.53%. During the construction of the Hi-C sequencing, a total of 380,119,286 raw paired-end reads were generated, which facilitated the anchoring of 96.24% of the draft genome into 7 pseudo-chromosomes (2n = 14) using the 3d-DNA software. The strong intra-chromosomal interaction signal indicated that the Hi-C assembly was of high quality, as shown in Figure S2. The final genome assembly of *R. roxburghii* was 531 Mb, which was at the chromosome level. Orthologs for 91.7% of the Complete Single-Copy BUSCOs were found in the *R. roxburghii* genome assembly.

### Genome annotation and whole genome duplication

Transposable elements (TEs) accounted for approximately 61.41% of the *R. roxburghii* genome, and 38.88% of these TEs were long terminal repeat (LTR) elements (Fig. 1B, Table S1). The *R. roxburghii* genome was predicted to contain 45,226 coding gene locis after masking repeat elements (Fig. 1B, Table S2). Orthologs for 90.1% of the Complete Single-Copy BUSCOs were found in the *R. roxburghii* annotation (Table S3), indicating that the annotated genome was largely complete. By aligning protein sequences with the Nr, Uniprot, KEGG, swissprot, TAIR and KOG databases obtained the annotation of 38,768, 37,887, 9,344, 28,264, 24,200 and 197 genes with best matches, respectively. A total of 7,417 genes could be mapped to KEGG pathways. The corresponding Gene Ontology (GO) annotation information for 27,451 genes was predicted based on the protein family information recorded in the UniProt database. Additionally, the InterProScan software was used to predict the Pfam and InterPro domain information for 22,056 and 37,057 genes, respectively (Table S4).

**Fig. 2:**
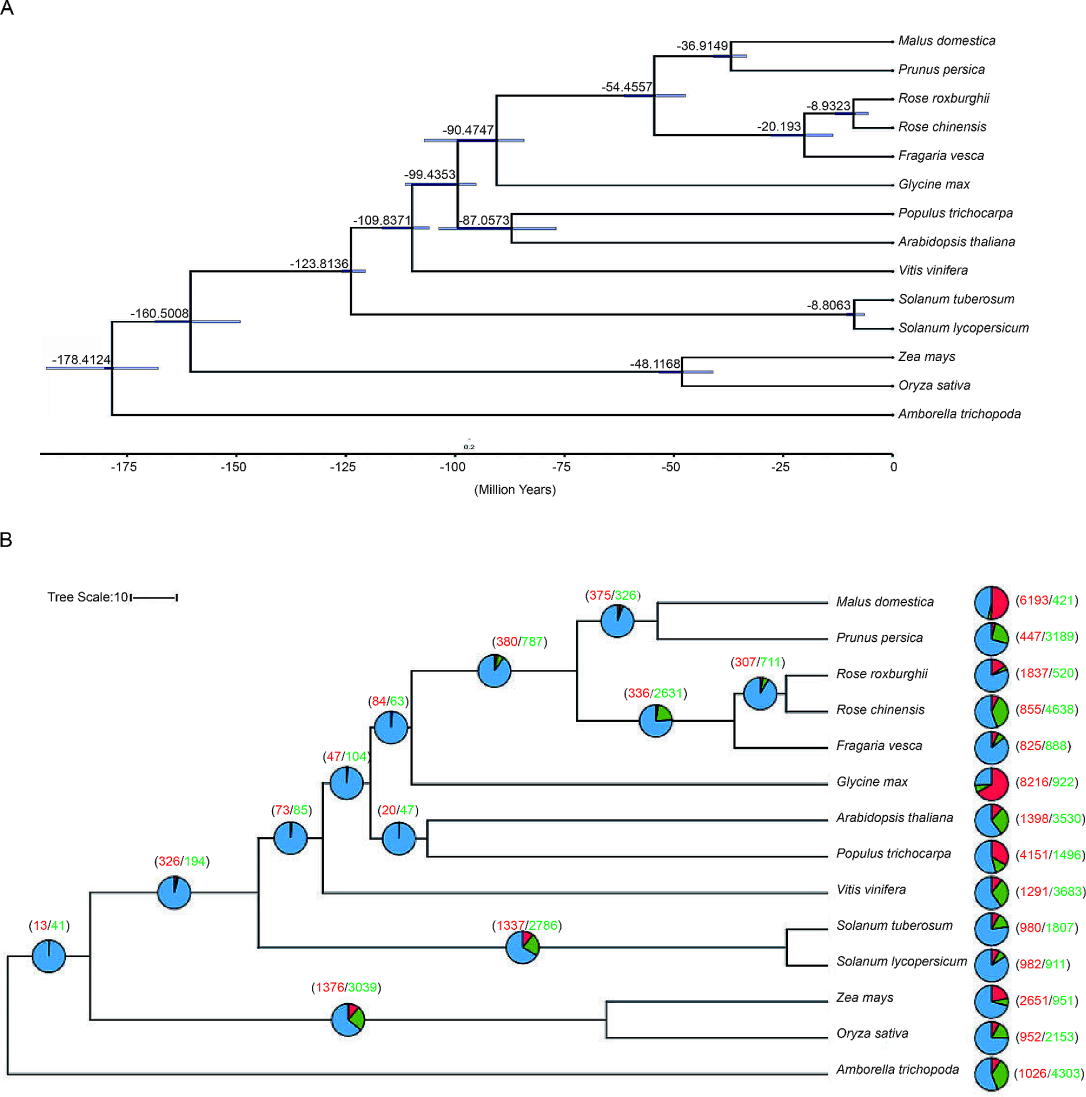
*R. roxburghii* phylogeny and gene family variation. (**A**) Phylogenetic tree based on single copy genes from 14 plant species showed divergence time. The numbers represented estimated divergence times. (**B**) Variation in the number of gene families relative to the ancestral node. In the pie chart, the red color represented the number of expanded gene families, the green color represented the number of contracted gene families, and the blue color represented the number of gene families with no change

**Fig. 3:**
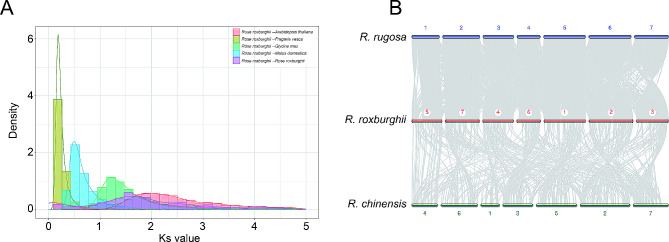
The whole genome duplication event and macro-collinearity analysis. (**A**) The synonymous substitutions per synonymous site (KS) distributions of orthologous and paralogous genes among *A. thaliana*, *F. vesca*, *G. max, M. domestica* and *R*. *roxburghii*. (**B**) Macro-collinearity analysis among *R. roxburghii*, *R. rugosa* and *R. chinensis*. The numbers in the chart represented chromosome identifiers

Orthologous protein groups from 14 angiosperms were delineated, yielding a total of 37,126 orthologous groups that included 453,632 genes. By analyzing the divergence time between *R. roxburghii* and other species, we found that it diverged from *Rose chinensis* approximately 5.58$\sim$13.17 million years ago (mya) (Fig. 2A). Our analysis indicated that there were 1,837 gene families expanded and 520 gene families contracted in the *R. roxburghii* lineage (Fig. 2B). GO enrichment analysis of the expanded gene families revealed significant enrichment of terms related to diterpenoid biosynthetic process, defense response, enzyme activities in multiple function (Table S5). Similarly, KEGG enrichment analysis of the expanded gene families showed significant enrichment of terms associated with sesquiterpenoid and triterpenoid biosynthesis, steroid hormone biosynthesis, toluene degradation etc. (Table S6). Ks analysis indicated that *R. roxburghii* shared the eudicot-specific WGT event with any species during its evolutionary process, but no recently WGD occurred (Fig. 3A). Previous studies had shown that *Rosa rugosa* and *Rosa chinensis* did not undergo species-specific whole-genome duplication events [52]. We conducted macro-collinearity analysis between *R. roxburghii* and *Rosa chinensis*, and the results indicated a conserved syntenic relationship between their chromosomes (Fig. 3B), further supporting the absence of recently WGD in *R. roxburghii*.

### Construction of co-expression network

**Fig. 4:**
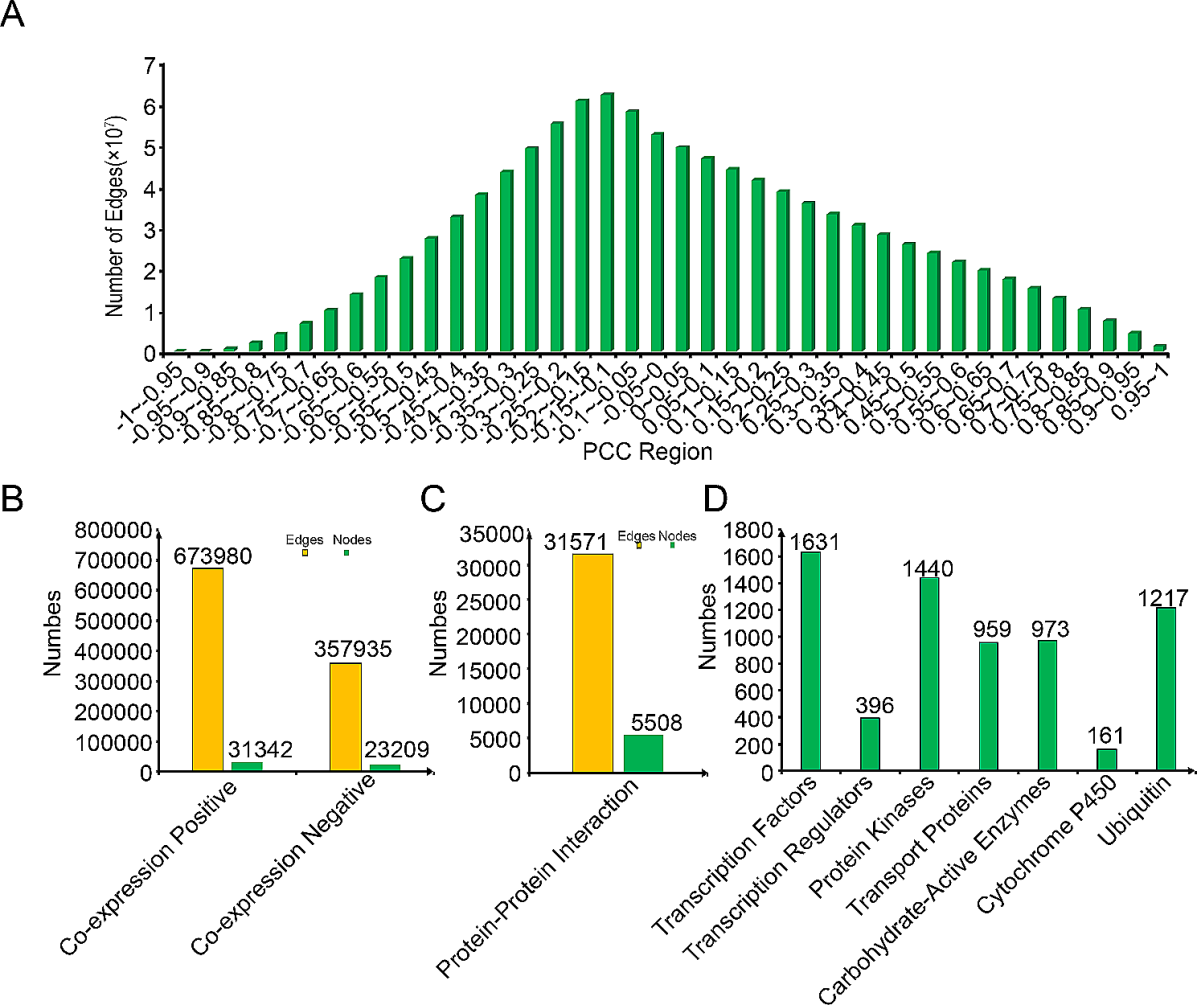
Network construction and Gene family classification. (**A**) The relationship between Pearson correlation coefficient (PCC) and the number of edges in the co-expression network. (**B**) Statistical analysis of nodes and edges in the positive co-expression network, negative co-expression network, and (**C**) Protein-Protein Interaction (PPI) network. (**D**) Gene family classification information available

Transcriptome data including 33 samples was obtained from the SRA database in NCBI. These samples included two sets of project data (The SRA numbers are SRP448410 and SRP167314). The SRP448410 was used to study 18 samples of calcium absorption of *R. roxburghii* seedlings. The SRP167314 was 15 transcriptome samples from different tissues. These RNA-seq datasets were mapped to the reference genome, resulting in a mapping ratio exceeding 80% (Table S7). We examined the distribution of Pearson correlation coefficient (PCC) values derived from the expression profiles. Most gene pairs exhibited either no correlation or weak correlation in terms of their expression patterns (Fig. 4A). To identify gene pairs with strong proximity within every two gene networks, we employed the MR (Matural Rank) approach based on their PCC ranking values.

Positive co-expressed genes sharing common expression patterns likely contribute to analogous biological processes. Assessment of these shared biological processes can be achieved by GO annotations. The co-expression network’s reliability is heightened with greater similarity in the GO annotation of gene pairs. To bolster the trustworthiness of our constructed network, we integrated a pre-existing gene set grounded in Gene Ontology (GO) terms, specifically selecting 636 GO terms characterized by gene counts ranging from 4 to 20. We conducted mutual predictions of GO between co-expressed gene pairs. We used whether GO could be accurately predicted as input for a specific binary classifier and calculated the true positive rate (TPR) and false positive rate (FPR), then plotted the ROC curve. The larger the Area Under the Curve (AUC) values in the ROC curve, the higher the true positive rate (TPR) for accurately predicting GO, and the more reliable the co-expression network. Comparing AUC values for different MR (Mutual Rank) values with PCC (Pearson Correlation Coefficient) > 0.7, we observed the maximum AUC when MR < 40, prompting the establishment of a network threshold of PCC > 0.7 and MR < 40 for the positive co-expression network (Figure S3). The thresholds for the negative co-expression network were set at PCC<-0.7 and MR < 40. The co-expression network of *R. roxburghii* consisted of 673,980 gene pairs in the positive co-expression network and 357,935 gene pairs in the negative co-expression network (Fig. 4B).

### Protein–protein interaction network

By predicting the orthologous genes between *Arabidopsi*s and *R. roxburghii*, we mapped the protein-protein interaction (PPI) network of *Arabidopsis* onto *R. roxburghii*, and 31,571 pairs of PPI relationships, involving a total of 5,508 genes were identified (Fig. 4C).

### DEGs in different transcriptome

In order to incorporate gene co-expression and protein-protein interaction (PPI) networks with gene expression data, we performed differential expression analysis on the transcriptome data. SRP448410 descripted that seedlings were transferred to a 2 mmol-L-1 Ca (CH3COO)2 absorbent solution for cultivation. Throughout the calcium starvation period (0 min), rapid calcium uptake period (5 min), and calcium saturation period (6 h), the roots and leaves of R. roxburghii seedlings were sampled for transcriptome sequencing. We compared the transcriptomes between 15 groups at different time points and identified differentially expressed genes. SRP167314 includes transcriptome samples of root, leave, flower, young fruit (YF), and mature fruit. We compared 10 different transcriptomes and identified differentially expressed genes. Finally, we obtained 25 differentially expressed gene (DEG) groups (Table S8).

### Gene family classification

We utilized the iTAK software to conduct analysis of transcription factors (TFs), transcription regulators (TRs), and protein kinases (PKs) in *R. roxburghii*. This analysis identified a total of 1,631 potential TFs, 396 TRs, and 1,440 PKs. Next, we employed a hidden Markov model (HMM) derived from the ubiquitin-proteasome dataset in the iUUCD v2.0 database to predict 1,217 genes participated in ubiquitin-proteasome system. Furthermore, based on the orthologous relationship between gene family specific database, we successfully identified 973 transprot protein coding genes, and 959 CAZy family genes. In addition, by KEGG annotation, we predicted 161 cytochrome P450 genes (Fig. 4D). These analysis provided valuable insights into the transcriptional regulation, protein kinase activity, ubiquitin-proteasome system in *R. roxburghii*.

### Database content

To enhance gene functional analysis in *R. roxburghii*, a comprehensive database called RroFGD has been developed (http://www.gzybioinformatics.cn/RroFGD). RroFGD consists of seven sections, namely Home, Network, Pathway, Tools, Gene Family, Download, and Help, each designed to improve usability and provide valuable insights for researchers. The Network section allowed access to both PPI and co-expression networks, enabling a deeper understanding of the intricate molecular interactions within *R. roxburghii*. To visualize the integration of these networks and DEGs (differentially expressed genes), a joint display node had been created. Within the network display, up-regulated DEGs were highlighted in red, while down-regulated DEGs were indicated in blue. This color-coded representation allowed for a clear distinction between the different expression patterns exhibited by the DEGs within the network.

Pathway section primarily consisted of gene annotations from the KEGG database. By clicking on the corresponding pathway, users could obtain the coding genes of all key enzymes in corresponding pathway. The Gene Family section encompassed various protein families, including CYP450, TF, TR, PK, TP, Ubiquitin and GAZy. These sections provided researchers with a comprehensive suite of tools for efficient gene functional analysis.

The Search tool allowed users to obtain interesting genes by using keywords, precise genes, transcript, or protein accession numbers. The Blast tool facilitated the screening of nucleic acid or protein sequences, identifying similarities within our database. GSEA (Gene Set Enrichment Analysis) provided an inclusive approach to gene set enrichment analysis. The Extract Sequence tool allowed for quick retrieval of gene sequences based on accession numbers and locations. Additionally, the Heatmap Analysis tool visually presented gene expression data, facilitating the interpretation of candidate gene lists. The integration of JBrowse provided an intuitive visualization of genomic and transcriptomic features, enhancing overall data exploration.

Download section provided convenient access to relevant information, ensuring easy retrieval of necessary resources. Furthermore, the help section offered a comprehensive user manual, guiding researchers how to use the RroFGD effectively.

### Identification and analysis of key enzyme genes in ascorbate biosynthesis

**Fig. 5:**
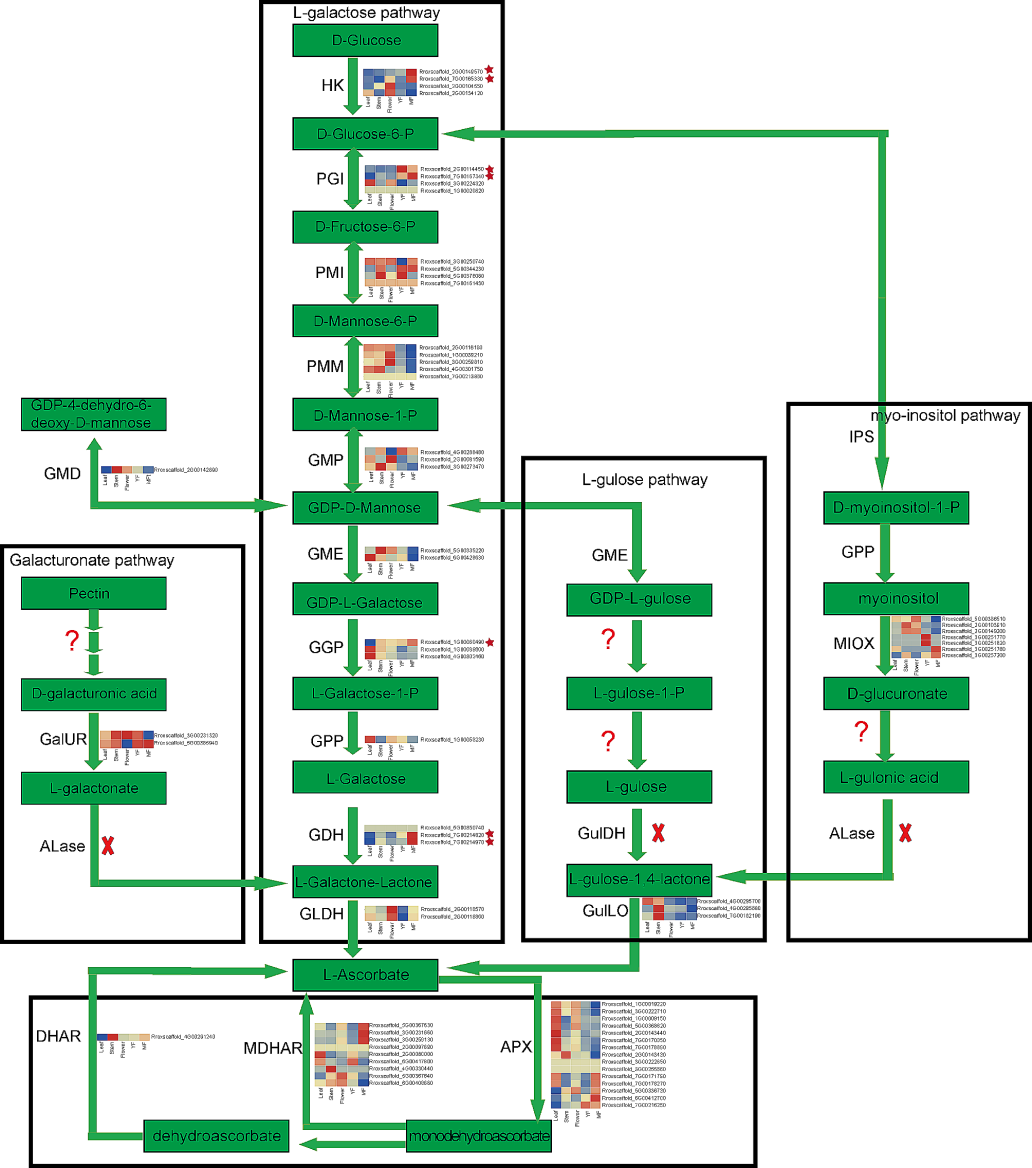
L-ascorbate biosynthesis and expression heatmap of key enzyme genes in *R. roxburghii*. The red cross represented key enzymes that were not annotated in the current genome based on the corresponding gene, the red question mark represented key enzymes on the current pathway that were still unclear, and the red star represented key enzymes that were significantly higher expression in mature fruits compared to other tissues

First, we identified key enzyme genes related to ascorbate biosynthesis by previous search [53, 54], such as *PGI* and *PMI*. Subsequently, we filtered these genes based on KEGG annotation information of *R. roxburghii* and further confirmed their relevance according to functional annotation information. We identified 69 key enzyme genes involved in ascorbate biosynthesis (Table S9), including 4 *HK*, 4 *PGI*, 4 *PMI*, 5 *PMM*, 3 *GMP*, 2 *GME*, 3 *GGP*, 1 *GPP*, 3 *GDH*, 2 *GLDH*, 9 *MDHAR*, 1 *DHAR*, 15 *APX*, 1 *GMD*, 2 *GalUR*, 3 *GulLO*, and 7 *MIOX* (Fig. 5, Table S9). Additionally, transcriptomic data analysis based on different tissues (leaves, stems, flowers, immature fruits, mature fruits) revealed the expression patterns in different tissues. Previous study showed that the mature fruits of *R. roxburghii* had the highest content of vitamin C [54]. Transcriptomic data analysis revealed that some key enzyme genes were significantly higher expression in mature fruits compared to other tissues, including 2 *HK*, 2 *PGI*, and 1 *GGP* (Fig. 5).

**Fig. 6:**
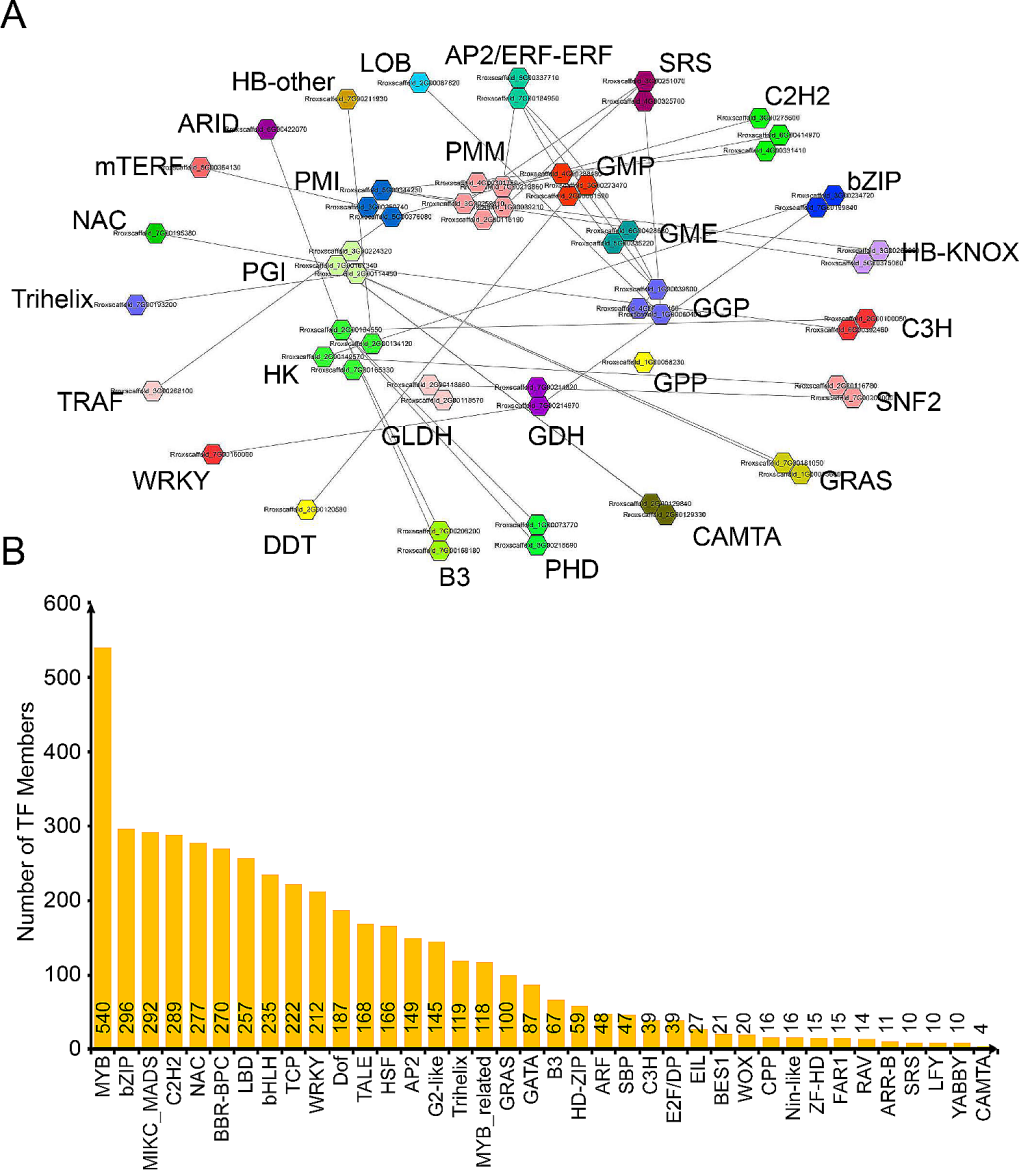
Gene co-expression network and cis-element analysis for key enzyme in L- ascorbate biosynthesis. (**A**) Gene co-expression network analysis of key enzyme genes in L-ascorbate biosynthesis. The dots in the inner circle represented key enzymes, while the dots in the outer circle represented transcription factors. (**B**) The number of transcription factor binding sites presented in the 1 kb promoter region of key enzymes coding genes. The horizontal axis represented transcription factors, and the vertical axis represented the number of transcription factor binding sites

By co-expression network analysis, we analyzed the regulatory genes of key enzyme genes involved in L-ascorbate biosynthesis and found that these key enzyme genes exhibited co-expression relationships with transcription factors, including AP2/ERF-ERF, SRS, C2H2, bZIP, HB-KNOX, C3H, SNF2, GRAS, CAMTA, PHD, B3, DDT, WRKY, TRAF, Trihelix, NAC, mTERF, ARID, HB-other, LOB, and so on (Fig. 6A). Analysis of transcription factor binding sites in the 1 kb region of these key enzyme promoters demonstrated many binding sites for transcription factors, including 11 co-expressed transcription factor families. The analysis results were partially consistent with the co-expression analysis results (Fig. 6B).

### Functional analysis of the *RrGDH* gene

**Fig. 7:**
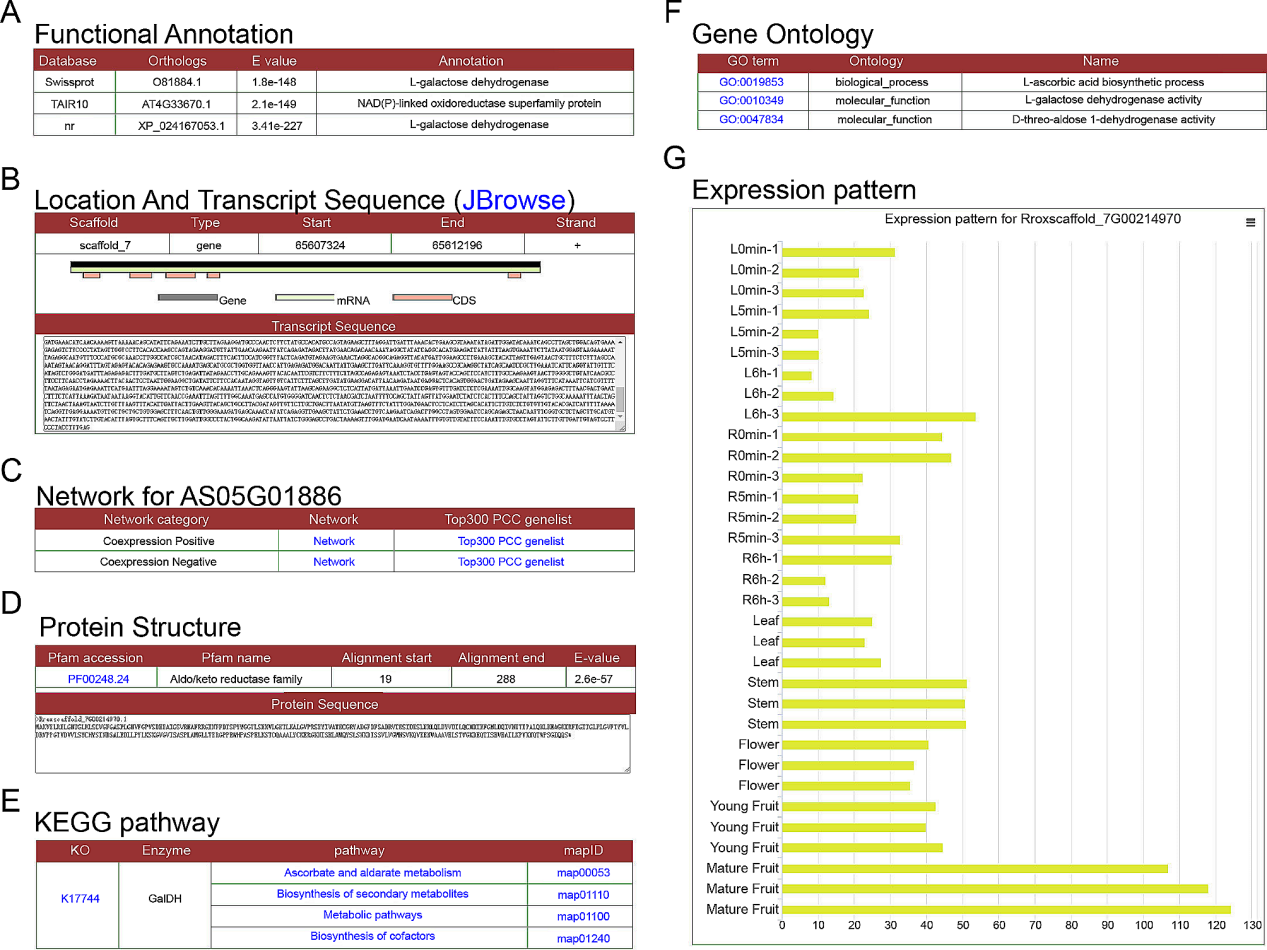
Gene details of *RrGDH* in RroFGD. (**A**) Functional annotations. (**B**) Location and transcript sequences. (**C**) Links for network. (**D**) Protein structure. (**E**) KEGG pathway. (**F**) GO annotation and (**G**) Expression pattern of RrGDH

**Fig. 8:**
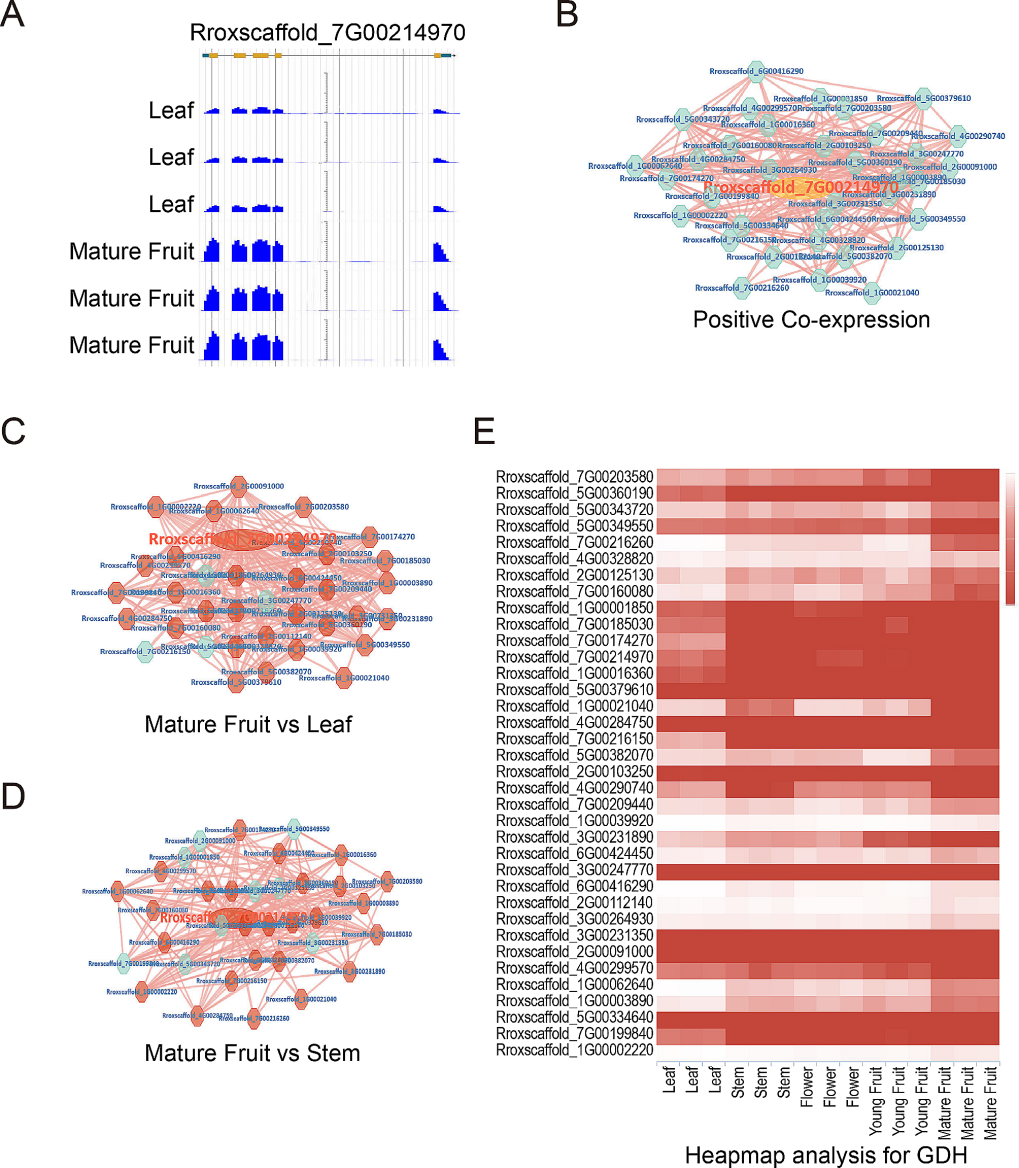
Expression and co-expression network analysis of *RrGDH*. (**A**) Expression level of *RrGDH* gene expression in leaf and mature fruit showing by JBrowse. (**B**) Positive co-expression gene network of *RrGDH*. The largest elliptical point represented the RrGDH gene. (**C**) Analysis of gene differential expression in the positive co-expression network when comparing mature fruit and leave transcriptomes. (**D**) Analysis of gene differential expression in the positive co-expression network when comparing mature fruit and stem transcriptomes. (**E**) Comparative analysis of *RrGDH* co-expressed genes expression in different transcriptome samples using heatmap analysis tool. The horizontal axis represented different tissues, while the vertical axis represents co-expressed genes of *RrGDH*

GDH (Glutamate dehydrogenase) is one of the key enzymes involved in the synthesis of L-ascorbate [55, 56]. A search based on RroFGD could provide detailed gene information for GDH. The gene Rroxscaffold_7G00214970 in *R. roxburghii* was identified as a member of L-galactose dehydrogenase (Fig. 7A), located on chromosome 7 from 65,607,324 to 65,612,196 bp with transcript sequence (Fig. 7B). Network links were also provided (Fig. 7C). It was found that Aldo/keto reductase family domain was located at protein sequence of GDH (Fig. 7D). KEGG annotation suggested that enzymes involved in L-ascorbate and aldarate metabolism and biosynthesis of secondary metabolites (Fig. 7E and F). Studies had also shown that mature *R. roxburghii* fruits had the highest content of vitamin C (VC) [54]. According to expression profiling analysis, we found that the expression level of GDH was higher in mature fruit compared to leaves (Fig. 7G). The display of reads mapping by JBrowse also revealed higher expression in leaves and stem (Fig. 8A). The expression of GDH showed a similar trend to the biosynthesis and accumulation of active compounds. Therefore, the analysis results suggested that GDH might be involved in the accumulation of L-ascorbate biosynthesis.

Furthermore, we conducted a co-expression analysis of *RrGDH* with its expression profiles. Network analysis revealed 35 genes that showed positive co-expression with *RrGDH* (Fig. 8B). Additionally, many genes in the co-expression network were significantly upregulated in the mature fruit when compared with leaves or stems (Fig. 8C, D and E). Therefore, our analysis suggested that the *RrGDH* gene played an important role in regulating biosynthesis of L-ascorbate.

## Discussion

*R. roxburghii* is a promising plant endemicity to the southern of China with medicinal and nutritional value. In recent years, the function of *R. roxburghii* has received wide attention and investigation [2, 10]. However, the genome information is still unclear. In our study, we constructed a high-quality *R. roxburghii* genome and then clustered the contigs into seven pseudochromosomes according to Hi-C data. A total of 90.1% genes were examined by BUSCO, which indicated that genome annotation had relatively high integrality. This is of great significance for the further study of gene function and genetic variation of *R. roxburghii*. At the same time, this study also provides an important resource for analysis of the evolutionary history, genome structure and identification of functional genes. However, despite the remarkable progress, there are still some challenges, such as how to better deal with heterozygous genomes and improve the precision and continuity of assembly. Future work will continue to focus on this aspect to better interpret the genomic characteristics of *R. roxburghii* and its relationship to plant morphology and biological function. *R. roxburghii* belongs to the Rosaceae family, and its genome information provides genetic resources for the future comparative genomic analysis of the Rosaceae family. Similar to other Rosaceae plants, the *R. roxburghii* we reported has not undergone species-specific whole-genome duplication (WGD) events. Therefore, like grapes, *R. roxburghii* can be used as a comparative subject to determine whether other species have undergone whole-genome duplication.

*R. roxburghii* contains numerous active components, especially a high content of L-ascorbate [54]. Based on the known biosynthetic pathway of L-ascorbate, we analyzed the synthesis pathway in *R. roxburghii* and identified 69 candidate genes, which might participate in L-ascorbate biosynthesis. The analysis of public expression profile data indicated that certain genes, including 2 *HK* (Rroxscaffold_2G00149570, Rroxscaffold_7G00165330), 2 *PGI* (Rroxscaffold_2G00114450, Rroxscaffold_7G00167340) and 1 *GGP* (Rroxscaffold_1G00060490), exhibited significantly higher expression in mature fruits compared to other tissues. Due to the high content of L-ascorbate in mature fruits, these genes could serve as candidate genes in L-ascorbate biosynthesis for further research. According to the current version of the genome, only the L-galactose biosynthetic pathway was complete. While the Galacturonate, L-gulose, and myo-inositol pathways lacked the corresponding key enzymes, such as ALase for the Galacturonate and myo-inositol pathways, and GulDH for the L-gulose pathway. Additionally, there was no significant expansion of key enzymes in L-ascorbate biosynthesis. Therefore, the reason for the significantly higher L-ascorbate content in *R. roxburghii* remained to be further explored.

To fully utilize the genome resources of *R. roxburghii*, we developed a functional genomics database named RroFGD, aiming to provide researchers with a wide range of resources and tools to gain deeper insights into the functional genes and related biological processes. To further elucidate gene functions of *R. roxburghii*, our database offered various analysis tools. These tools contained gene set enrichment analysis (GSEA), local alignment search tool (Blast), extract sequence, heatmap and JBrowse. Users could use these tools according to their research needs to uncover the biological significance within the co-expression network. To facilitate effective utilization of the database, we provided the detailed usage example that demonstrated how to analyze functional genes in RroFGD.

The establishment of co-expression network and protein interaction network not only contributed to understand the interaction between genes, but also lay an important foundation for further study of the biological function and regulatory mechanism of *R. roxburghii*. By integrating with co-expression networks and offering various analysis tools along with detailed usage examples, we are committed to *R. roxburghii* research and provide valuable resources for researchers in related fields. Currently, the database relies on existing gene expression datasets, and how to ensure data quality and coverage remains a challenge. In the future, we plan to expand the scale and diversity of the dataset to provide more comprehensive and accurate analysis results. Additionally, we aim to refine and expand the analysis tools and functionalities of the database, involving continuous I mprovement and updating the latest advancements in the field of *R. roxburghii*.

The identification of differentially expressed genes helps us to understand the gene expression changes of *R. roxburghii* in different growth stages and different tissues. Especially in transcriptome samples from calcium absorption experiments, our analysis revealed that many gene expressions changed significantly, which might reflect the plant’s response to changes in calcium content in the environment. In transcriptome analysis of different tissues, we found differences in gene expression between roots, leaves, flowers, young and mature fruits. This gave us clues to further understand the functional differences between the different organs of *R. roxburghii*. The identification of the differentially expressed genes laid an important foundation for us to further explore the biological characteristics, functional genes and genes related to environmental response and adaptation of *R. roxburghii*. In addition, our study conducted a detailed classification and identification of the *R. roxburghii* gene family, and the results of these analyses provided important clues for us to further understand the genomic function and regulatory mechanism. The identification of transcription factors, transcription regulators and protein kinases contributed to understanding of the transcriptional regulatory network of genes. The prediction of ubiquitin-proteasome system provided important information for understanding protein degradation and regulation. The identified gene family and cytochrome P450 gene also provided important references for understanding the metabolic pathway and biosynthesis process of active ingredients in *R. roxburghii*.

Overall, in our research, we exhibited a high-quality genome of *R. roxburghii*. We further identified 69 candidate genes for the biosynthesis pathway of active compound of L-ascorbate. In order to facilitate usage, we constructed a functional database RroFGD for researchers to mine more gene function of *R. roxburghii*. The genomic resources and RroFGD will provide a solid foundation for the future research about *R. roxburghii*.

### Electronic supplementary material

Below is the link to the electronic supplementary material.

Supplementary Material 1

Supplementary Material 2

## Data Availability

The raw data of our project have been deposited in the Sequence Read Archive (SRA) in National Center for Biotechnology Information (NCBI) (Project ID: PRJNA1025299). The genome assembly and gff3 file reported in this paper have been deposited in the Genome Warehouse [57] in National Genomics Data Center [58], Beijing Institute of Genomics, Chinese Academy of Sciences/China National Center for Bioinformation, under accession number GWHEROQ00000000.
