# Supplementary material for: The chromosome-level genome and functional database accelerate research about biosynthesis of secondary metabolites in Rosa roxburghii
Source: BMC Plant Biol. 2024 May 17;24:410. doi: 10.1186/s12870-024-05109-1 (PMC11100184; doi:10.1186/s12870-024-05109-1)
Supplement: Supplementary file 2 — Supplementary Material 2 [file 12870_2024_5109_MOESM2_ESM.docx]

***Supplementary Material***

**The chromosome-level genome and functional database accelerate research about biosynthesis of secondary metabolites in *Rosa roxburghii***

**Supplementary Figures**


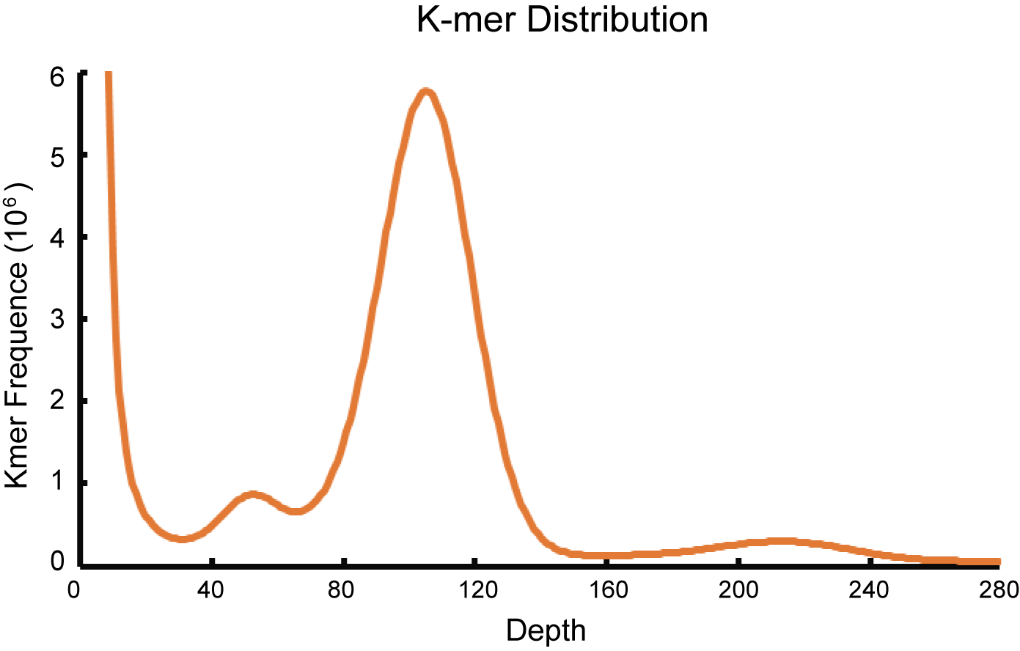


**Figure S1.** Genome survey of *R. roxburghii* using 19 k-mer distribution analysis.


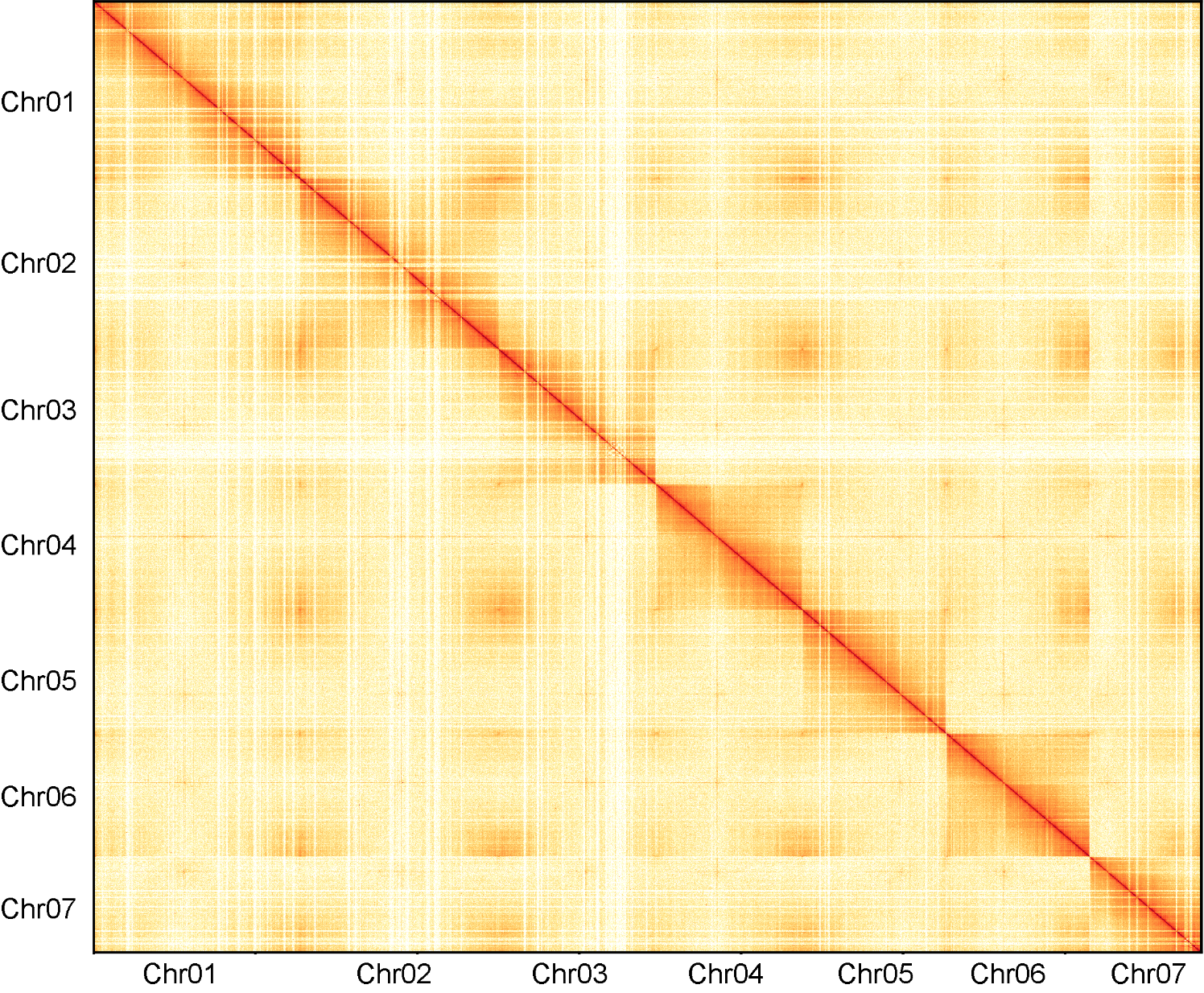


**Figure S2.** Hi-C intrachromosomal correlation heatmap for the genome assembly.


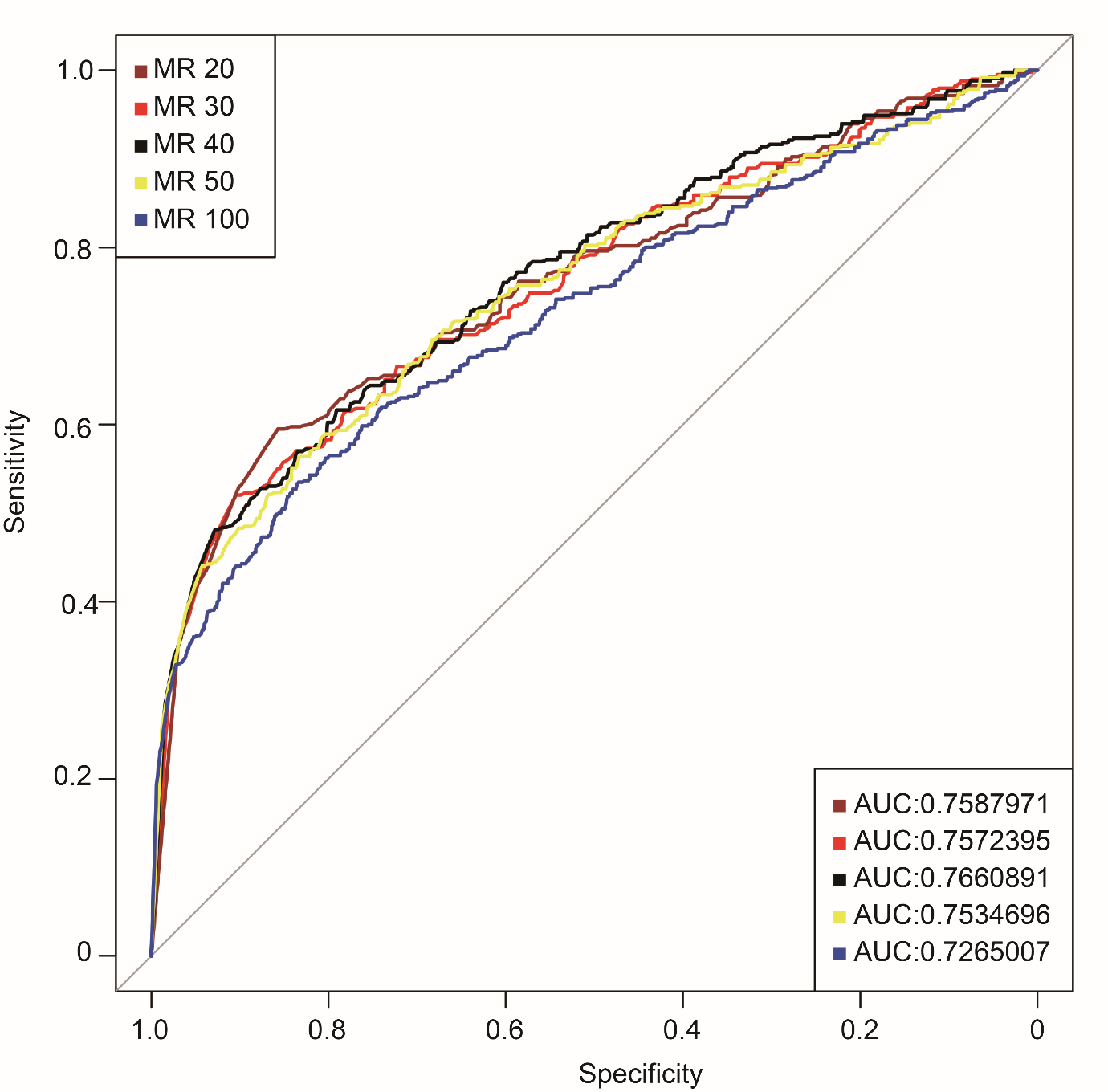


**Figure S3.** The AUC value of the co-expression network under different MR values when PCC>0.7.
